# Genome sequence of *Enterococcus gallinarum* AH4, a milk oligosaccharide-degrading strain isolated from suckling rats

**DOI:** 10.1128/MRA.00395-23

**Published:** 2023-09-21

**Authors:** Yuji Yamamoto, Yasunori Suzuki, Reo Tsukuda, Chikara Asai, Masaki Ishizuka, Yuji Tsujikawa, Iwao Sakane, Ro Osawa, Takao Mukai

**Affiliations:** 1 Laboratory of Cellular Microbiology, School of Veterinary Medicine, Kitasato University, Aomori, Japan; 2 Laboratory of Animal Hygiene, School of Veterinary Medicine, Kitasato University, Aomori, Japan; 3 Central Research Institute, ITOEN, Ltd., Shizuoka, Japan; 4 Graduate School of Agricultural Science, Kobe University, Hyogo, Japan; Wellesley College, Wellesley, Massachusetts, USA

**Keywords:** milk oligosaccharide, *Enterococcus gallinarum*, suckling rats, sialyllactose, genome sequence, symbiosis

## Abstract

We had previously isolated *Enterococcus gallinarum* AH4, a strain capable of degrading rat milk oligosaccharides. In this study, we determined the whole-genome sequence of AH4. This whole-genome information will expand our understanding of milk oligosaccharide-mediated symbioses between bacteria and host mammals.

## ANNOUNCEMENT

Human milk oligosaccharides are selectively utilized by specific species of bifidobacteria, fostering the development of a bifidobacteria-dominant gut microbiota (1). However, such bacteria–host symbioses have rarely been reported in other mammals. We previously isolated *Enterococcus gallinarum* AH4, a strain that degrades 3’- and 6’-sialyllactose (the major milk oligosaccharides in rats), from the intestinal contents of suckling rats using an enrichment culture with 3’-sialyllactose, in contrast to the *E. gallinarum* type strain, which is unable to utilize these oligosaccharides (2). *E. gallinarum* was also implicated as a contributor to the development of rat infants’ gut microbiota through its ability to degrade 3’- and 6’-sialyllactose (3). The whole-genome information of *E. gallinarum* AH4 determined in this study will expand our understanding of milk oligosaccharide-mediated symbioses between bacteria and host mammals.

To obtain genomic DNA, an overnight culture of M17 broth (Becton, Dickinson and Company, NJ, USA) supplemented with 0.5% glucose grown from a single colony was inoculated into the same medium at 2% and incubated anaerobically at 37°C to log phase. Bacterial cells were then suspended in Solution I (50 mM glucose, 25 mM Tris-HCl, 10 mM EDTA, pH 8.0). RNase A (1 mg/mL, Sigma-Aldrich), lysozyme (1 mg/mL, Sigma-Aldrich) and mutanolysin (125 U/mL, Sigma-Aldrich) were added to the suspension and incubated for 30 minutes at 37°C. Sodium dodecyl sulfate (0.5%) and proteinase K (0.1 mg/mL, Wako Pure Chemicals, Osaka, Japan) were added to the suspension, which was then incubated at 37°C overnight. Genomic DNA was purified using standard phenol/chloroform extraction (4) and an Agencourt AMPure XP Kit (Beckman Coulter, CA, USA).

Whole-genome sequencing was performed using NovaSeq (Illumina, San Diego, CA, USA) and PacBio Sequel (Pacific Biosciences) sequencers. For NovaSeq sequencing, an index-tagged library was prepared using a VAHTS Universal Pro DNA Library Prep Kit (Illumina). DNA shearing was performed using a Covaris Focused-ultrasonicator S220 (Covaris, LLC, USA), and 150-bp paired-end reads were sequenced. The NovaSeq raw reads were trimmed using CLC genomics workbench v22.0.2 (Qiagen, Hilden, Germany) with default settings. The DNA library for PacBio Sequel was prepared using g-TUBE (Covalis) and a SMRTbell Express template preparation kit v2.0 (Pacific Biosciences), with a target length of 10 kb. Sequences were analyzed on the PacBio Sequel platform, and adapter sequences were trimmed using the FASTX-Toolkit (http://hannonlab.cshl.edu/fastx_toolkit/). Hybrid assembly of NovaSeq and PacBio Sequel-trimmed reads was performed using Unicycler v0.4.8 with default parameters (5). The contig sequences were annotated using DFAST v1.6.0 with default parameters (https://dfast.nig.ac.jp/) (6).

The assembled genome consists of 11 contigs with a total length of 3,686,711 bp (Table 1). Considering that the genome size of the *E. gallinarum* standard in the NCBI database ranges from 3.18 to 3.82 Mbp, the data is presumed to represent almost the entire length of the chromosome.

**TABLE 1:** Assembly statistics and general genome information

Sequence and genome information	Data for *E. gallinarum* AH4
Assembly and genome statistics	
NovaSeq sequencing	
No. of reads	6,753,952
Total no. of bases	1,013,092,800
After trimming with CLC Genomics Workbench	
No. of reads	6,753,807
Total no. of bases	1,007,735,542
PacBio sequel sequencing	
No. of reads	172,317
Total no. of bases	560,373,203
Read length N50 (bp)	3,669
Coverage (x)	425
Chromosome features^*a*^	
Total sequence length (bp)	3,686,711
No. of sequences:	11
Longest sequences (bp)	2,042,355
N50 (bp):	2,042,355
Gap ratio (%)	0
GC content (%)	40.3
No. of CDSs^*b*^	3507
Average protein length (aa)	302.9
Coding proportion (%)	86.4
No. of rRNAs	10
No. of tRNAs	59
No. of CRISPR regions	0

^*a*^ All genomic statistics are output from the DFAST pipeline.

^*b*^ CDSs, coding sequences.

## Data Availability

This genome sequence has been deposited in DDBJ/ENA/GenBank under accession numbers BSYC01000001-BSYC01000011. The raw data were deposited in the DDBJ Sequence Read Archive (DRA) under the accession number DRA016189, and the accession numbers for the Illumina and PacBio sequence data were DRR462666 and DRR462665.
